# Ca^2+^-ATPase Molecules as a Calcium-Sensitive Membrane-Endoskeleton of Sarcoplasmic Reticulum

**DOI:** 10.3390/ijms22052624

**Published:** 2021-03-05

**Authors:** Jun Nakamura, Yuusuke Maruyama, Genichi Tajima, Yuto Komeiji, Makiko Suwa, Chikara Sato

**Affiliations:** 1 Health and Medical Research Institute, National Institute of Advanced Industrial Science and Technology (AIST), Central 6, 1-1-1 Higashi, Tsukuba, Ibaraki 305-8566, Japan; yuusuke.maruyama@gmail.com (Y.M.); y-komeiji@aist.go.jp (Y.K.); 2 Institute for Excellence in Higher Education, Tohoku University, 41 Kawauchi, Aoba-ku, Sendai, Miyagi 980-8576, Japan; g_tajima@tohoku.ac.jp; 3 Biological Science Course, Graduate School of Science and Engineering, Aoyama Gakuin University, 5-10-1 Fuchinobe, Chuou-ku, Sagamihara, Kanagawa 252-5258, Japan; suwa@chem.aoyama.ac.jp

**Keywords:** ryanodine receptor, Ca^2+^-ATPase, two-dimensional crystallization, ATP, calcium, membrane endoskeleton, transmission electron microscopy, cell morphology, cell dynamics

## Abstract

The Ca^2+^-transport ATPase of sarcoplasmic reticulum (SR) is an integral, transmembrane protein. It sequesters cytoplasmic calcium ions released from SR during muscle contraction, and causes muscle relaxation. Based on negative staining and transmission electron microscopy of SR vesicles isolated from rabbit skeletal muscle, we propose that the ATPase molecules might also be a calcium-sensitive membrane-endoskeleton. Under conditions when the ATPase molecules scarcely transport Ca^2+^, i.e., in the presence of ATP and ≤ 0.9 nM Ca^2+^, some of the ATPase particles on the SR vesicle surface gathered to form tetramers. The tetramers crystallized into a cylindrical helical array in some vesicles and probably resulted in the elongated protrusion that extended from some round SRs. As the Ca^2+^ concentration increased to 0.2 µM, i.e., under conditions when the transporter molecules fully carry out their activities, the ATPase crystal arrays disappeared, but the SR protrusions remained. In the absence of ATP, almost all of the SR vesicles were round and no crystal arrays were evident, independent of the calcium concentration. This suggests that ATP induced crystallization at low Ca^2+^ concentrations. From the observed morphological changes, the role of the proposed ATPase membrane-endoskeleton is discussed in the context of calcium regulation during muscle contraction.

## 1. Introduction

The sarcoplasmic reticulum (SR) plays a primary role in regulating cytoplasmic calcium concentrations in skeletal muscle cells [1,2,3]. After ryanodine receptor (RyR) channels release Ca^2+^ from the SR to contract the muscle [3], Ca^2+^-ATPase (110 kDa), an integral membrane protein [4,5], relaxes it again by transporting cytoplasmic calcium ions into the SR lumen, which lowers the cytoplasmic Ca^2+^ concentration [1,2]. To achieve this the Ca^2+^-ATPase undergoes an ATP-dependent *E*_1_ (high affinity state for calcium)–*E*_2_ (low affinity state for calcium) transition [6,7,8,9]. Several studies of SR in vertebrates show (i) that the SR membrane system mainly consists of two regions of bulbous terminal cisternae and tubular longitudinal elements [10,11]; (ii) that the terminal cisterna is associated with the transverse tubules via bridging structures comprised of RyR channels (calcium-release channel of the SR) and referred to as ”feet” [12,13,14]; and (iii) that the tubular elements of the SR pass through the Z line of myofibrils [10,11,15]. The SR structure prompts the hypothesis [11] of a single SR network in a muscle fiber. The longitudinal tubules are the predominant part of the SR [10,11], and the ATPase proteins comprise about 90% of the total proteins in it [16,17]. The ATPase molecule is asymmetrically distributed in the membrane with about 2/3 of its mass exposed to the cytoplasm [4,5]. The cytoplasmic domain of the molecule is visualized as a surface particle (~40 Å in diameter by negative-staining electron microscopy) on the SR membranes [18,19,20]. The intramembranous domains of the ATPase revealed by freeze fracture of the membrane look like ~85 Å particles in the electron microscope [21,22,23]. The average particle density of the 40 Å domains on the SR surface (particles/µm^2^) is 3 to 4 times larger than the particle density of the 85 Å particles, suggesting that the ATPase molecules have a tetrameric structure in the membrane [18]. However, it has been shown that in the absence of ATP, the ATPase molecules have predominantly disordered disposition and form random contacts with each other within the membrane [23]. Is tetramer formation necessary for their Ca^2+^ pump activity? It is unnecessary, because ATPase molecules monomerized with detergent still function as calcium pumps in the presence of ATP [24,25]. However, in the absence of ATP, monomerized ATPase molecules have been shown to bind calcium in a different way to the two membranous ATPase conformers [26,27]. This implies the existence of various functional conformations of the ATPase molecules [27]. Based on the observed heterogeneous calcium binding of the membranous ATPase molecules without ATP, the ATPases have been hypothesized to exist as two conformational variants (the A and B forms) of the same amino acid sequence, with a ratio of 1:1 in the SR membrane [26,27,28]. Moreover, a tetramer model was suggested for these hypothesized molecules, based on the enzymatic reaction of Ca^2+^-ATPase with acetyl phosphate [29,30], which drives its Ca^2+^ transport like ATP [31].

To investigate the disposition of the Ca^2+^-ATPase molecules, we have investigated SR vesicles isolated from rabbit skeletal muscle using negative staining and transmission electron microscopy (TEM) while regulating the experimental conditions, including the concentrations of ATP and Ca^2+^ and temperature, without using any inhibitor.

## 2. Results

**Figure 1:**
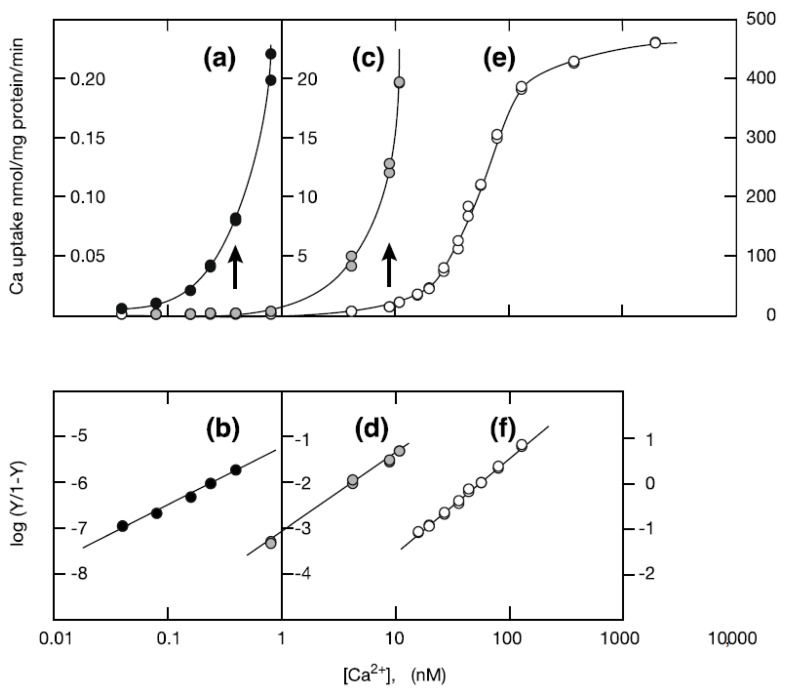
Calcium dependence of Ca^2+^-transport activity in the 0.04 nM–9.9 µM Ca^2+^ concentration range and the change of the Hill coefficient; the Hill coefficient is a parameter that indicates the degree of the cooperativity in binding calcium ions at the Ca^2+^-ATPase. (**a**,**c**,**e**) Calcium dependence of the transport activity. **(b**,**d**,**f**) The corresponding Hill plots. *Y* is the ratio of the transport activity at each calcium concentration to the maximum activity (445 nmol Ca/mg of protein/min). (**a**,**b**) 0.04–0.81 nM Ca^2+^ (*black filled circles*). (**c**,**d**) 0.04–11 nM Ca^2+^ (*grey circles*). (**e**,**f**) 0.04 nM–9.9 µM Ca^2+^ (*unfilled circles*). The ordinates of Ca^2+^-transport activity and log (Y/1-Y) shown at the left, middle and right Y-axes in this figure, correspond to their values in (**a** and **b**), (**c** and **d**), and (**e** and **f**), respectively.

The calcium transport reaction of SR Ca^2+^-ATPase molecules is coupled to large conformational changes in the molecule [32,33]. Moreover, it has been suggested that the ATPase *E*_1_–*E*_2_ transition that takes place, is accompanied by a monomer-dimer transition [34]. To see if there is an orderly disposition of the molecules in the presence of ATP (23 mM), we prepared SR under conditions expected to minimize movement of the ATPase molecules, i.e., at an extremely low concentration of Ca^2+^ (0.9 nM or less) and at 0 °C. The low calcium concentration in the presence of ATP seems to be comparable to the calcium state (<0.03 µM of Ca^2+^) [35] of cytosol in myocytes in the resting state. Under this condition, it is thought, as reported earlier [36], that almost all of the ATPase molecules are in the *E*_1_ state (high affinity state for calcium); the *E*_1_ state of the molecules with ATP and without calcium seems to be a standby state before the start of the calcium-triggered, calcium transport reaction. At this calcium concentration, the Ca^2+^-transport activity (~0.2 nmol of calcium/mg of protein/min) was observed to be less than 1/2000 of the maximum (~450 nmol of calcium/mg of protein/min) (Figure 1). Assuming that SR vesicles are spherical and based on literature reports that the diameter [37] and ATPase density [23] of SR vesicles are 0.15 µm and 31–34,000/µm^2^ respectively, the total number of the ATPase molecules in a vesicle is calculated to be 2200–2400. Since the ratio of active ATPase molecules (phosphorylatable molecules with ATP) to the total number of molecules is reported to be 0.5 [38], 1100–1200 molecules in such a vesicle are expected to be in an active form that precedes the calcium transporting state. Taken together, at the extremely low 0.9 nM Ca^2+^ concentration employed, the number of the active molecules in operation is roughly estimated to be less than 1 molecule/vesicle; the remaining molecules seem to be a standby state, as mentioned above.

It is important to know how reliable the observed value of the Ca^2+^-transport activity is at such a low calcium concentration. To evaluate the transport activity, we measured the calcium dependence of the activity at calcium concentrations ranging from 0.04 nM to 9.9 µM (Figure 1a,c,e). The dependence can be described by three Hill plots [39] with different slopes (Hill coefficients) (Figure 1b,d,f), as follows. (i) In the range 16–130 nM Ca^2+^ (Figure 1f), the Hill coefficient of the slope was about 2.1, consistent with the established concept of cooperative binding of two calcium ions to the ATPase [6,7,8,9]. (ii) In the range 0.80–11 nM Ca^2+^ (Figure 1d), the value was about 1.7, i.e., a little smaller than 2, but still close to 2. (iii) In the range 0.04–0.4 nM Ca^2+^ (Figure 1b), the value was lower and only about 1.3, which is much less than 2. We conclude that the reliability of Ca^2+^-transport activity measurements made at around 0.9 nM Ca^2+^ and the higher concentration (16–130 nM) of Ca^2+^ is comparable, but uncertain in the lower concentration range of 0.04–0.4 nM.

**Figure 2:**
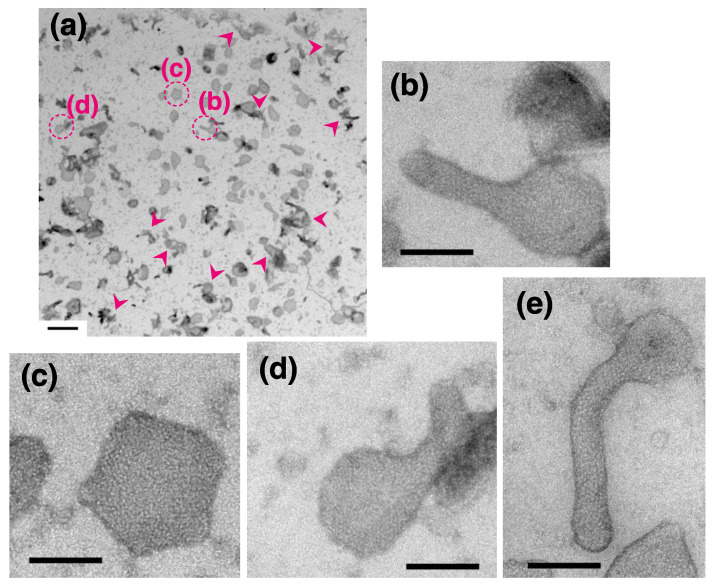
SR vesicles in the presence of ATP and 0.02 nM Ca^2+^ at 0 °C. (**a**) Overview showing the SR vesicle population within the 5.4 µm by 5.4 µm specimen grid area imaged by TEM. Variously shaped vesicles and agglomerates are present. Some agglomerated vesicles are indicated by arrowheads as a reference. (**b**–**d**) Higher magnification images of the tightly elongated (**b**), pentagonal (**c**) and crookedly elongated (**d**) vesicles marked with the dotted circles in (**a**). A crystalline array of 40 nm particles is clear in (**b**). (**e**) A crookedly elongated vesicle, not present in overview (**a**). Scale bar in (**a**): 0.5 µm. Scale bars in (**b**–**e**): 100 nm.

Subsequent TEM observation revealed that when incubated overnight in the presence of 23 mM ATP at 0 °C (see Section 4 for details) some of the SR vesicles agglomerated and formed clumps (Figure 2a, arrowheads). To obtain meaningful results, a vesicle population without any apparent agglomeration was selectively imaged. In contrast, in the absence of ATP, no significant vesicle agglomeration was observed, as in agreement with earlier previously reported [18,19,20] (see details of how these vesicles are prepared without ATP as described later in method section).

**Figure 3:**
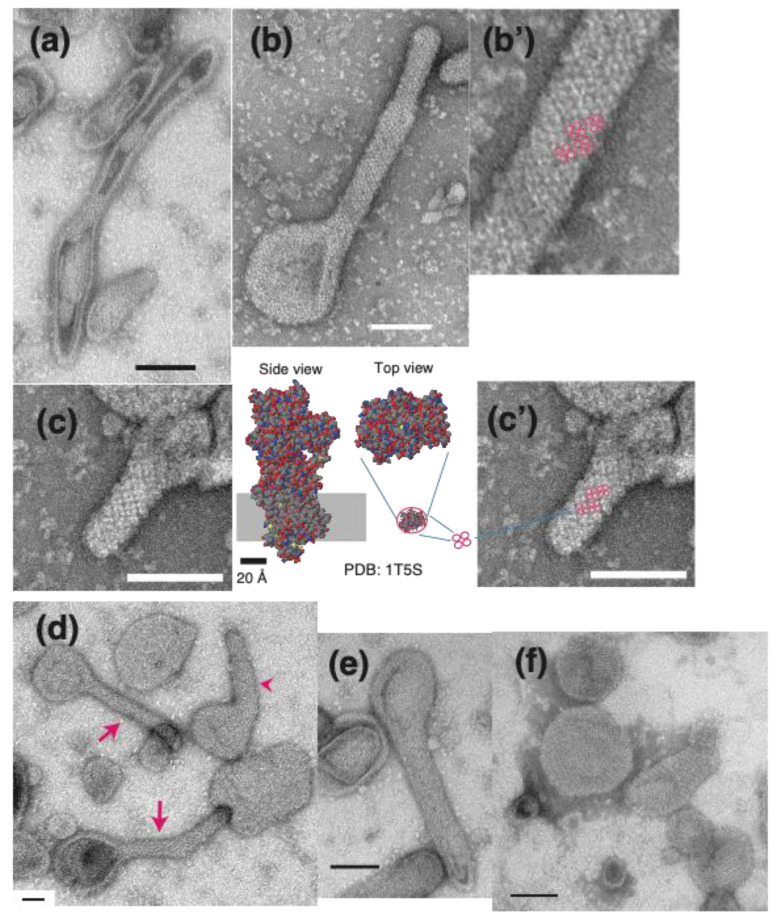
Typical images of the tightly elongated SR vesicles at 0.9 nM Ca^2+^ or less at 0 °C. The SR vesicles were classified as with or without a crystal-array of surface particles. With a crystal array (**a**) at 0.04 nM and (**b**,**c** and **d** (arrows)) at 0.9 nM Ca^2+^. Without a crystal array (**d** (arrowhead) and **e**) at 0.9 nM Ca^2+^. When a crystal-array was difficult to distinguish the vesicle was classified as ‘without a crystal-array’. In (**b’**) (enlargement of a region in **b**) and (**c**), clear tetramer units of the surface particles are marked with circles. A model of a rabbit Ca^2+^ ATPase monomer in the *E*_1_ state with bound calcium (PDB 1T5S) [40] is shown for comparison. (**f**) Decavanadate-induced crystalline array of surface particles in the SR vesicles, as a reference; the SR preparation (1.0 mg of protein/mL) was incubated with 100 mM imidazol-HCl (pH 7.4) containing 0.12 M KCl, 5 mM MgCl_2_ and 0.5 mM Na-decavanadate under the conditions of 0.02 nM Ca^2+^ at 0 °C overnight. Scale bars in (**a**–**c**,**c’**,**e** and **f**): 100 nm. Scale bar in (**d**): 50 nm.

At less than 0.9 nM Ca^2+^ (0.02 nM) with 23 mM ATP at 0 °C, crystalline arrays (like latticework and/or a ladder) of vesicle-surface particles (~40 Å diameter) were observed in some tightly elongated (tadpole-shaped or straight tube-like) vesicles (Figure 2b), pentagonal vesicles (Figure 2c) and crookedly (bent) elongated vesicles (Figure 2d,e) (see the legend of Figure 2 for details). We first focused on the tightly elongated vesicles. For example, a crystalline array of the 40 Å particles is easily distinguishable in one elongated vesicle and covers an area larger than 32 nm × 46 nm (Figure 2b). Figure 3 shows typical images of tightly elongated vesicles with (Figure 3a–d, arrows) and without (Figure 3d, arrowhead, and Figure 3e) a crystalline array (see the legend of Figure 3 for details). Imaging at higher magnification clearly revealed that the individual 40Å particles in the crystalline arrays are tetramers (Figure 3b,c); an atomic model of a rabbit Ca^2+^ ATPase monomer in the *E*_1_ state with bound calcium (PDB: 1T5S) (PDB:1T5S) [40] determined by X-ray crystallography was shown for comparison. In crystalline regions of the vesicles, almost 90 × 90 Å tetrameric units (particles) arrange themselves in rows and chains to form the crystalline network (clearly visible tetramer units are marked with circles in Figure 3b,c). This observation substantiates the tetramer model suggested for the ATPase structural unit [29,30], originally proposed by Scales & Inesi [18]. In contrast to tightly elongated vesicles, pentagonal vesicles basically only included crystalline arrays at their periphery, whereas the center of the pentagonal vesicles was not well crystalized (Figure 2c).

In the SR preparation employed here, Ca^2+^-ATPase forms a high percentage (~90%) of the total protein [41] (see Section 4). According to earlier studies of particles (~40 Å diameter) of SR ATPase molecules [18,19,20], the observed 40 Å particles on the SR vesicles are thought to be the cytoplasmic part of the ATPase molecule (Figure 3c). To obtain morphological evidence for this hypothesis, the SR preparation was treated with decavanadate (an inhibitor of the ATPase) [42,43,44,45]; decavanadate-induced ribbons of Ca^2+^-ATPase dimers are a specific feature of the surface particles of rabbit SR Ca^2+^-ATPase molecules. Decavanadate-induced crystallization of the 40 Å particles was also observed in the SR vesicles employed here (Figure 3f), as reported earlier [42,44], indicating that they are ATPase. However, the crystalline arrays formed did not predominantly include ATPase-dimer like units; in contradiction to the earlier observations [45], tetramer-like units were also distinguished. The whole surface of the SR vesicles was covered by the 40 Å surface particles comprised of ATPase molecules. The ATPase proteins are embedded in phospholipid membranes [7]. It has been shown that the ATPase molecule (110 kDa) is asymmetrically distributed in the SR membrane with ~2/3 of its mass on the cytoplasmic surface of the membrane and ~1/3 of its mass in the membrane [4,5] (Figure 3c’ left model [40]). In the SR preparations employed here, the ATPase protein accounts for about 90% of the total protein mass of the membrane [41]. In agreement with the large cytoplasmic part of the ATPase and the high density (31–34,000/µm^2^ [23]) of this protein in the membrane, the whole surface of the vanadate-treated SR vesicles was covered by 40 Å particles, as shown in Figure 3f. As to the inhibition mechanism of decavanadate, it had been suggested that the ATPase is in the *E*_2_ state [34]. On the other hand, it has been reported that thapsigargin (TG) (another inhibitor of the ATPase, different from decavanadate) stabilizes the ATPase in the *E*_2_ state without inducing ATPase crystallization [44], though TG promotes the vanadate-induced crystallization [44,46]. Therefore, the mechanism of the vanadate-induced crystallization remains to be solved.

**Figure 4:**
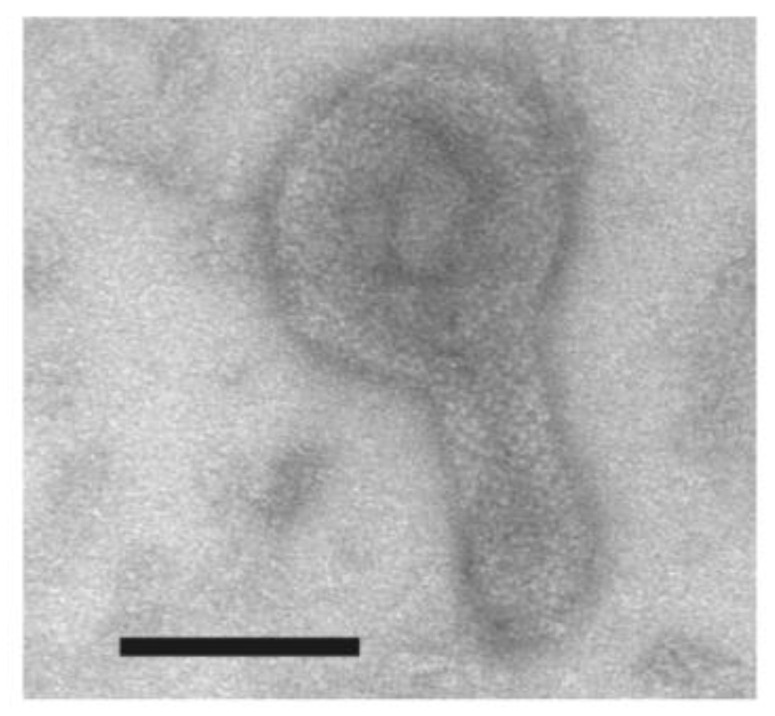
The SR vesicle with a crystal-array of surface particles, observed in the presence of 5 mM ATP at 25 °C. The SR preparation (0.3 mg of protein/mL) was incubated with 5 mM ATP for 1 min under the conditions of 0.04 nM Ca^2+^ at pH 7.4 and 25 °C. Scale bar: 100 nm.

The decavanadate-induced crystalline array like two rails of a railway track (Figure 3f), seems to be entirely different to the network of the tetramer chains formed by the ATPase at low calcium concentrations in the presence of ATP (compare Figure 3b–d (indicated by arrow) with Figure 3f). In contrast, the tetramer network seems to be fairly close to the monomer latticework of the surface particles, formed in a buffer containing calcium and lanthanide ions but without ATP [34]. Crystalline arrays of tetramers were also observed in the elongated SR vesicles in the samples with 5 mM ATP at 25 °C (Figure 4), although the tetramer unit of the surface particles was not very clear in the array. These observations suggest that crystallization of the surface ATPase particles occurs under the physiological conditions of ATP concentration and temperature.

To describe the morphological differences between vesicles at various Ca^2+^ concentrations ≤ 2.0 µM in the presence of ATP, we classified the vesicles imaged by TEM into seven types according to their elongation and surface particle array as follows (Table 1). The vesicles were first classified as ‘elongated’ or ‘round’ (see footnote of Table 1). In further rounds, the elongated vesicles were classified as the ‘tightly elongated’ or ‘crookedly elongated’ type, and the tightly elongated vesicles were further classified according to whether they had a crystalline array of 40 Å particles (Figure 3d, arrow) or not (Figure 3d, arrowhead; see figure legend of Figure 3 for details). The crystalline arrays sometimes covered a large area of the vesicle, as shown in Figure 2b and Figure 3a–d, arrow. Vesicles with a tubular tetramer crystalline array were relatively rare among four different Ca^2+^ concentrations examined here; only one of the 1225 vesicles examined had a cylindrical tetramer-based crystalline array at ≤ 0.9 nM Ca^2+^ (see the footnote of the Table 1). Vesicles with crystalline array (Figure 3b–d, arrow) were thus classified, regardless of the presence or absence of the cylindrically organized tetramers in the array. The vesicles with tetramer-based arrays were repeatedly detected in the independent experiments.

At low Ca^2+^ concentration ≤ 0.9 nM, the percentages of the elongated and round vesicles relative to the total number of vesicles were about 13.8% and 86.2%, respectively. The elongated vesicles were further classified into tightly elongated (4.5% (55 vesicles)) and crookedly elongated vesicles (9.3% (114 vesicles)). About half (31 vesicles) of the tightly elongated vesicles included a crystalline array.

**Figure 5:**
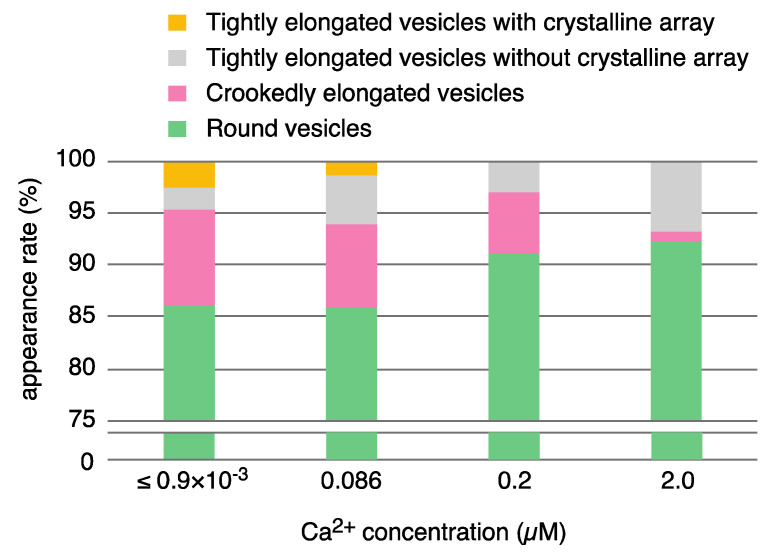
Bar plot showing the appearance rates of the various types of SR vesicle relative to the total number of vesicles imaged at ≤ 0.9 nM–2.0 µM Ca^2+^. The vesicle classification reported in Table 1 was simplified to four types: tightly elongated vesicles with a crystalline array (yellow), tightly elongated vesicles without a crystalline array (gray), crookedly elongated vesicles (pink), and round vesicles (green). The rates are represented by the average percentages of the number of each vesicle type relative to the total number of vesicles imaged at the respective calcium concentrations. As indicated, the full range below 75% is not shown for the round vesicles, for convenience.

**Figure 6:**
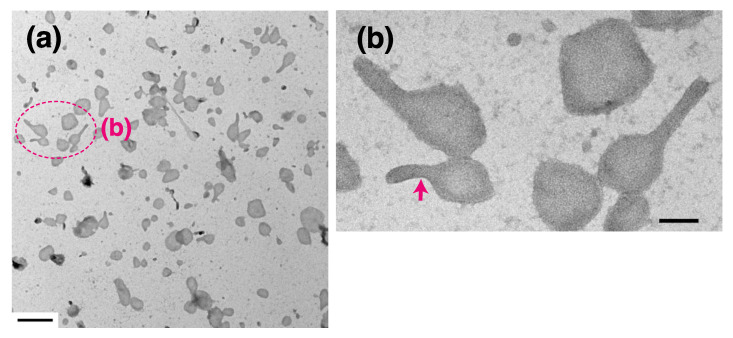
SR vesicles at 86 nM Ca^2+^ in the presence of ATP at 0 °C. (**a**) Low magnification image of the vesicles. (**b**) Higher magnification of the annotated area surrounding the tightly elongated vesicles (arrow). The vesicles have a crystal-array of particles. Scale bar in (**a**): 0.5 µm. Scale bar in (**b**): 100 nm.

**Figure 7:**
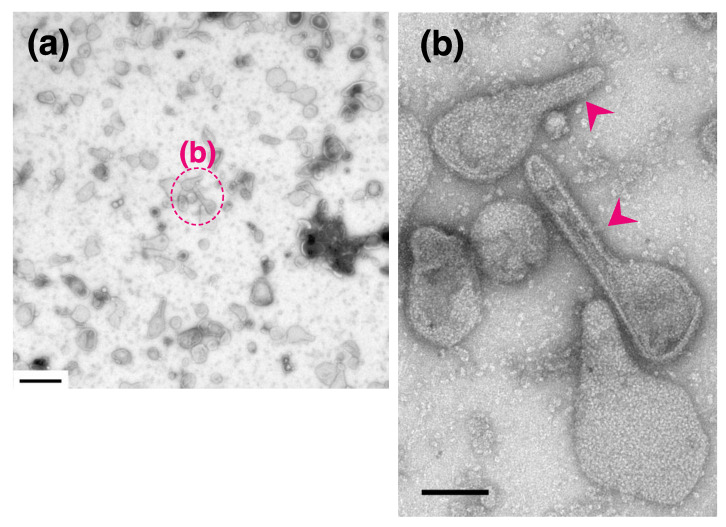
SR vesicles at 0.2 µM Ca^2+^ in the presence of ATP at 0 °C. (**a**) Low magnification overview image of the vesicles. (**b**) Higher magnification image of the annotated area surrounding some elongated vesicles. The tightly elongated vesicles (arrowheads) did not include a crystal-array of particles. Scale bar in (**a**): 0.5 µm. Scale bar in (**b**): 100 nm.

We found remarkable differences between the crystalline arrays of Ca^2+^-ATPase particles in vesicles incubated in buffer containing 86 nM, 0.2 µM or 2.0 µM Ca^2+^ in the presence of 23 mM ATP (Table 1 and Figure 5). The Ca^2+^-transport activity of the SR is about half-maximum at 86 nM Ca^2+^, and has almost reached its maximum at 0.2 µM Ca^2+^ (Figure 1 and see Ref. [36]), while muscle tension reaches its maximum at 2.0 µM Ca^2+^ [35]. At 86 nM Ca^2+^, elongated vesicles did not have tetramer- crystal-arrays, although some of the tightly elongated vesicles included a crystal-array (Figure 6a,b, dotted circles). At 0.2 µM Ca^2+^, no distinct crystal-arrays were found in the tightly elongated vesicles present (Figure 7a,b). These vesicles had a similar shape to vesicles with tetramer-based arrays (0.9 nM Ca^2+^ or less and ATP; Figure 3a–c) and possessed equally clear outlines. The results suggest that the absence of chains of tetramers at 86 nM Ca^2+^ and of the crystal-array itself at 0.2 µM Ca^2+^ might be due to the increased calcium concentration. Moreover, arrays were absent when most of the ATPase molecules were actively transporting calcium, i.e., at the saturating concentration of 0.2 µM Ca^2+^.

**Figure 8:**
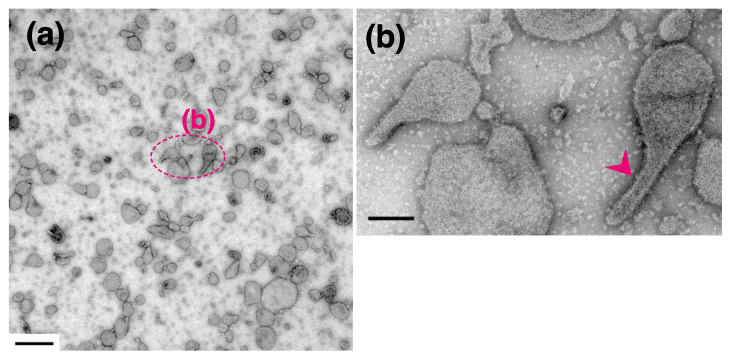
SR vesicles at 2.0 µM Ca^2+^ in the presence of ATP at 0 °C. (**a**) Low magnification image of the vesicles. (**b**) Higher magnification of the annotated area. The tightly elongated vesicles (arrowhead) did not include a crystalline array. Scale bar in (**a**): 0.5 µm. Scale bar in (**b**): 100 nm.

In the presence of ATP, the percentage of tightly elongated vesicles to the total number of vesicles was almost the same (4.5–6.8%) at all of the calcium concentrations examined (Table 1). It is noteworthy that the elongated state is maintained in the absence of a distinguishable crystal-array, even at high Ca^2+^ concentration (2.0 µM) (Figure 8a,b), when muscle tension reaches its maximum [35]. The elongated vesicles without any clear ATPase array, might reflect a change in the agglomeration state of the ATPase molecules during their Ca^2+^-transport reaction.

**Figure 9:**
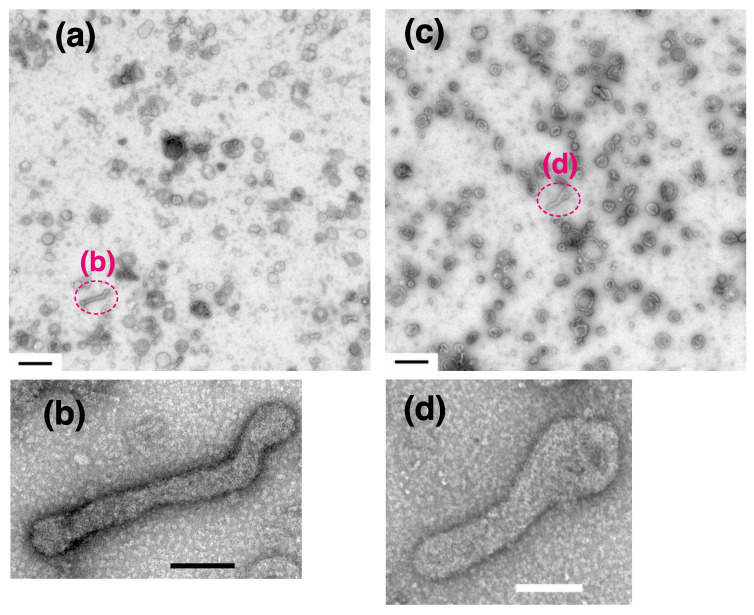
SR vesicles in the absence of ATP at 0 °C. The SR preparation was incubated in buffer containing 0.02 nM Ca^2+^ (**a**,**b**) or 2 µM Ca^2+^ (**c**,**d**). (**a**,**c**) Low magnification images. The vesicles did not agglomerate significantly in either buffer. (**b**,**d**) Higher magnification images of the annotated areas in the panels above. Round vesicles and crookedly elongated vesicles were observed, although tightly elongated vesicles were not imaged at all. Scales bars in (**a**) and (**c**): 0.5 µm. Scale bars in (**b**) and (**d**): 100 nm.

On the other hand, in the absence of ATP, almost all of the SR vesicles (~96%) were classified as the ‘round type’. At a low calcium concentration (0.02 nM) in the absence of ATP, cohesion of the vesicles was rare (Figure 9a), in contrast to the vesicles in the presence of ATP (Figure 2a). Almost all of the SR vesicles (~96%) were classified as the round type (Table 2). The round vesicles did not have an orderly disposition of particles, as reported earlier [18,19,20]. The remaining vesicles (~4%) belong to the ‘crookedly elongated type without orderly disposition of the particles’ (Figure 9a,b and Table 2). Similar results were obtained at higher calcium concentration (2 µM) in the absence of ATP (Figure 9c,d and Table 2).

**Figure 10:**
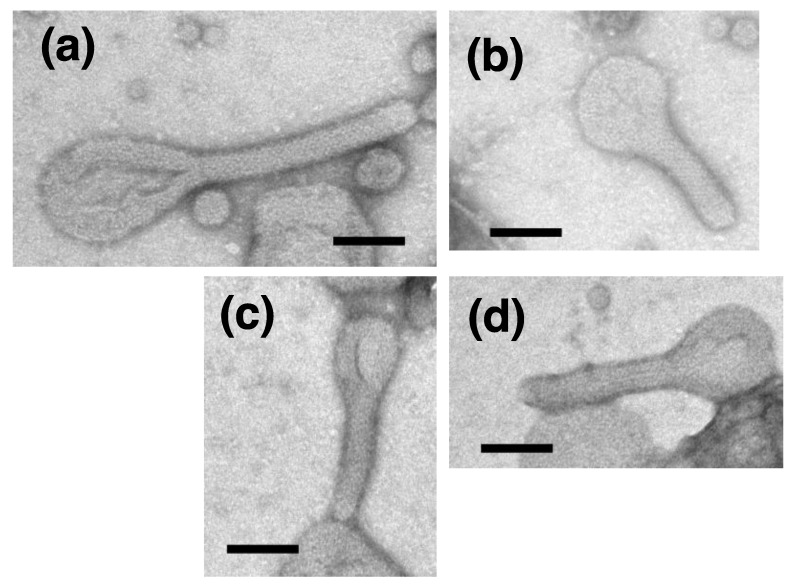
SR vesicles in the presence of the Ca^2+^-ATPase inhibitor, thapsigargin. The SR preparation (0.3 mg of protein/mL) was incubated with 100 mM imidazol-HCl (pH 7.4) containing 0.12 M KCl, 5 mM MgCl_2_, 5 mM EGTA and 23 mM ATP with and without TG at 0 °C overnight. (**a**,**b**) Vesicles in the presence of DMSO (0.09% (*v*/*v*)) without TG. Many vesicles had crystal-arrays of particles. (**c**,**d**) Vesicles in the presence of TG at the ratio of 5 nmol TG/mg SR protein, which is half the dose, required for completely inhibit the ATPase (see text for details). The vesicles did not have crystal-arrays. Thapsigargin disturbed crystal formation by the surface particles at 0.04 nM Ca^2+^. Scale bars: 100 nm.

**Figure 11:**
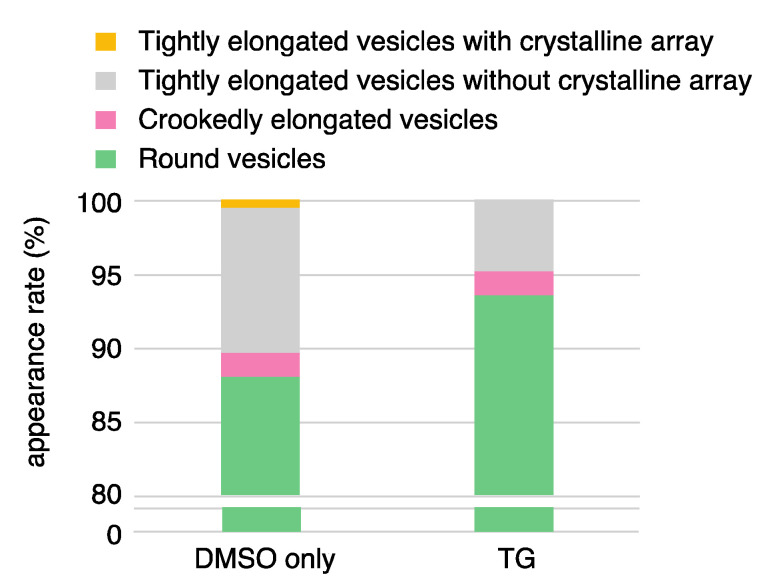
Bar plot showing the appearance rates of the various types of SR vesicle in samples with and without thapsigargin (TG). The rates are represented by the average percentages of the number of each vesicle type relative to the total number of vesicles. As indicated, the full range below 80% is not shown for the round vesicles, for convenience.

To clarify the relationship between the enzyme state (active or inactive) and the crystal-array state of Ca^2+^-ATPase, the effect of ATPase inactivation by TG (Ca^2+^-ATPase inhibitor) [44] on SR vesicles in a partly crystallized condition at low Ca^2+^ concentration ≤ 0.9 nM in the presence of ATP, was examined. We observed that the elongated vesicles extensively collapsed in the presence of TG (10 nmol TG/mg protein), which completely inhibits the Ca^2+^-ATPase activity [44]. To assess the critical dose, the TG concentration was lowered to 5 nmol TG/mg SR protein. This decrease prevented the crystal collapse to a certain degree. Because the TG employed was solubilized in dimethylsulfoxide (DMSO), the assay without TG was performed in the presence of DMSO (0.09% *v*/*v*). A crystal-array of the surface particles was observed in the tightly elongated vesicles (Figure 10a,b), like in the absence of DMSO (Figure 2b and Figure 3a–d). Thus, the DMSO ‘contamination’ did not seem to significantly affect the crystal formation by the particles. However, no SR vesicles with a clear crystal-array were observed in the presence of TG with DMSO (Figure 10c,d) (Table 3); the vesicles without clear array were classified as ‘vesicles without crystal array’, like the vesicles in Figure 3d (arrowhead) and Figure 3e (see figure legend in Figure 3 for details). The percentage of ‘tightly elongated vesicles with and without a crystal-array’ to the total number of vesicles was decreased to almost half (from 10.3 to 4.8%) by TG (Table 3 and Figure 11). Nevertheless, the data indicate that TG at least disturbs the ATP-induced crystallization of the ATPase molecules, supporting the above-mentioned hypothesis that the active ATPase molecules arrange themselves orderly into a crystal on the SR membrane with the help of ATP to elongate part of the vesicle. It should be noted that the disturbing effect of TG on the crystallization, is in contrast to the promoting effect of TG on the formation of decavanadate-induced dimer crystal arrays [44,46].

## 3. Discussion

We have established an experimental method by which Ca^2+^-ATPase molecules in the SR membrane are reorganized, in a highly reproducible manner, in response to Ca^2+^ concentration changes in the presence of ATP. The results suggest that some of the round SR vesicles present transform into tightly elongated vesicles that have a crystalline array of Ca^2+^-ATPase molecules, with the help of ATP at a low concentration of calcium where the ATPase molecules scarcely transport Ca^2+^ (Figure 3a–d). The crystalline array in the elongated vesicles gradually degraded (Figure 6 and Figure 7), as calcium concentration increased to 0.2 µM, the concentration at which the ATPase molecules fully perform their transport activities. Considering the fact that the transmembrane parts [4,5] of Ca^2+^-ATPase molecules form close, but disorderly contacts with each other in the absence of ATP [18,23], the observed correlation between the tight elongation and the crystalline array suggests that the Ca^2+^-ATPase molecules form a dynamic calcium-sensitive, membrane-endoskeleton to elongate the SR vesicles.

Recently, it has been reported that physiological concentrations (5–10 mM) of ATP may act as a biological hydrotrope [47], i.e., it can both prevent agglomeration and dissolve protein aggregates. The observed effect of ATP on the crystallization of the SR Ca^2+^-ATPase molecule is, therefore, possibly best described based on the hydrotrope model; ATP may transform the structure of SR Ca^2+^-ATPase molecules lacking ATP, into their intrinsic state to facilitate crystal formation in the isolated SR membrane. Such an ATP-induced transformation of the ATPase molecules has also been found in their calcium binding [36,48].

**Figure 12:**
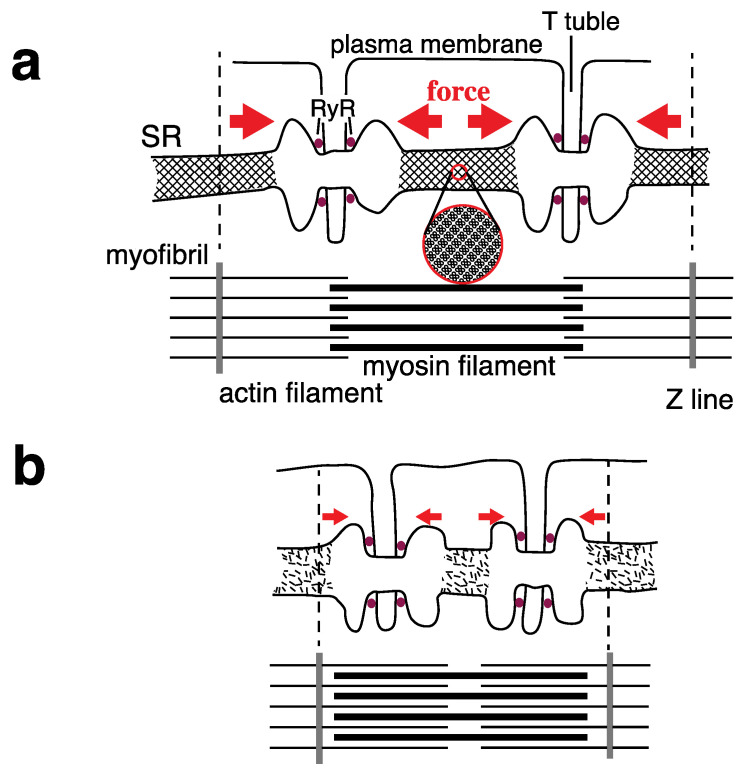
Schematic representation of the formation (**a**) and collapse (**b**) of a crystalline array of the Ca^2+^-ATPase surface particles in the SR of rabbit skeletal muscle, which link with the resting and contracting states of the muscle, respectively.

The data reported here suggests the existence of a relationship between crystal formation by Ca^2+^-ATPase molecules and the development of striated, SR pipe structures that require the stretching force, and might contribute to the regulation of RyR, as shown in Figure 12. Namely, in the SR of rabbit skeletal muscle in vivo, the formation and collapse of a crystalline array of Ca^2+^-ATPase molecules could link to the resting and contracting states of the muscle, respectively. Considering the pentagonal vesicle with a crystalline array of Ca^2+^-ATPase molecules (Figure 2c), we infer that SR might not only elongate but also expand when the ATPase molecules crystallize.

For the further analyses of the precise movement of the ATPase molecules, we are planning to perform statistical image analysis based on machine learning in combination with cryo-electron microscopy (cryo-EM) [49] and liquid-phase immuno-EM including the use of the atmospheric scanning electron microscope (ASEM) we have developed [50,51,52,53,54].

## 4. Materials and Methods

Isolation of the SR vesicles—The SR vesicles were isolated from the white skeletal muscle of adult male rabbit using the method of Weber, et al. [55] with some modification [41]. The white skeletal muscle was homogenized in 4 volumes of 0.1 M KCl containing 5 mM histidine (pH 7.5). The homogenate was centrifuged at 1000× *g* for 20 min, and the supernatant was centrifuged at 8000× *g* for 30 min to remove mitochondria. The supernatant was filtered through No. 5A filter paper. The filtrate was centrifuged at 30,000× *g* for 50 min. The pellets were then suspended in 30 volumes of 0.16 M KCl containing 5 mM Tris-maleate (pH 6.8) to remove contaminating myosin. This suspension was centrifuged at 35,000× *g* for 50 min. The pellets were again suspended in 0.12 M KCl containing 5 mM Tris-maleate (pH 6.8). This suspension containing SR fragments was stored at −80 °C with ~0.3 M sucrose. The SR preparation obtained has been shown to have a high content of the ATPase protein [41], being about 90% of the total protein of the SR membrane, which is characteristic of the longitudinal tubules of the SR [16,17], and has been confirmed to be of fast twitch muscle type [56]. The adult male albino rabbit (Japanese white rabbit; *Leporinae* *Trouessart*) was purchased from Oriental Yeast Co., Ltd. (Tokyo, Japan).

Electron microscopy—The SR preparation (0.3 mg of protein/mL) was incubated with 100 mM imidazole (pH 7.4), 0.12 M KCl, 5 mM MgCl_2_, 0.02 nM–2 µM Ca^2+^ with and without 23 mM ATP at 0 °C overnight, unless otherwise indicated. After overnight incubation of the SR with the ATP at 2 µM Ca^2+^, about 35% of the ATP was exhausted, and the pH of the SR suspension was lowered by 0.12–0.15. Calcium concentration was adjusted with ethylenebis (oxyethylenenitrilo) tetraacetic acid (EGTA) by taking into account the loss due to the formation of Ca-ATP and Mg-ATP; the association constants for Ca-EGTA, Ca-ATP and Mg-ATP were taken as 10.942 × 10^6^ [57], 8.241 × 10^3^ and 1.466 × 10^4^ M^−1^ [58]. Negative staining was performed as follows: SR vesicle solution was adsorbed to thin carbon films rendered hydrophilic by glow discharge in air and supported by copper mesh grids [59]. Samples were negatively stained with a 20 mg/mL uranyl acetate solution for 30 s twice, blotted, and dried in air. This procedure was carried out within a cooling box at 5 °C. The specimens were viewed with JEM-1230 (JEOL, Tokyo, Japan) and H 7600 (Hitachi, Tokyo, Japan) (Hitachi, Tokyo, Japan) transmission electron microscopes at 100 and 80 kV accelerating voltage, respectively.

Assay of Ca^2+^-transport activity—The transport reaction was carried out in a medium containing 0.05 mg of protein/mL SR, 40 mM Tris-maleate (pH 7.4), 0.12 M KCl, 5 mM MgCl_2_, 0.04 nM–9.9 µM ^45^CaCl_2_, and 5 mM K-oxalate with 5 mM ATP at 25 °C. Calcium concentration was adjusted with added calcium and EGTA; the association constant of Ca-EGTA, employed here was 12.274 × 10^6^ M^−1^ [57]. The reaction was initiated by the addition of ATP and was stopped by the addition of 50 mM EGTA with the same volume (0.5 mL) as the reaction medium. The reaction times with ATP were 20 min, 10 min and 30 s, respectively, at 0.04–0.16 nM, 0.24–1.64 nM and 0.40 nM–9.9 µM Ca^2+^. Aliquots (0.8 mL) of the stopped reaction medium with SR were filtrated through a nitrocellulose filter (0.45 µm pore size, TM-6, Advantec Toyo, Tokyo). Then, the SR on the filter was washed twice with 0.5 mL of a washing solution (0.2 mM EGTA, 20 mM Tris-maleate (pH 7.4), 5 mM MgCl_2_). Finally, the amount of ^45^Ca incorporated into the SR vesicles was measured.

## 5. Conclusions

The data obtained suggests the following: (i) At a resting concentration (<0.03 µM at pH 7.4) of Ca^2+^ for muscle contraction, crystal formations of the ATPase molecules are induced by ATP in the SR in muscle. (ii) The crystal could be further developed to three-dimensional cylindrical crystals, elongating the SR, which might cause a stretching force in the SR to set the RyRs (assumed to be a mechanoreceptor) on standby for depolarization of the muscle plasma membrane. (iii) The ATPase crystal is suggested to be collapsed by Ca^2+^ released through the RyRs in muscle, and stretching force of the SR could be lowered, which might result in the RyRs closing. Taking into account that the SR network passes through the Z line (a partition of myofibril) in a striated muscle fiber of vertebrates, SR might act as a calcium-regulated, mechanical apparatus to perform a united control of the enormous number of the RyRs within a muscle fiber, in addition to its role as a muscle relaxing apparatus. The model presented here sheds light on a fundamental property of protein other than functioning as enzyme, that is the property of “being sticky” (personal communication from Dr. Doolittle, R.F., University of California, 1983), which might contribute to the formation of ordered structures.

## Figures and Tables

**Table 1:** Classification of the SR vesicles observed at < 0.09 nM, 0.086, 0.2, and 2.0 µM Ca^2+^ in the presence of ATP. The observed vesicles (major axis > 0.065 µm) were classified as the elongated or round types, and then, the elongated vesicles were sub-classified as tightly elongated or crookedly elongated. The tightly elongated vesicles were further classified into the two types ‘with’ and ‘without’ a crystalline array of the 40 Å surface particles. For the analysis, 9–12 TEM images that included one or more tightly elongated vesicle with or without a distinct crystal-array, respectively, were recorded at each calcium concentration (<0.9 nM, 0.086 µM Ca^2+^, 0.2 µM Ca^2+^ and 2.0 µM Ca^2+^). For each concentration, the vesicle populations (56–228 vesicles) within each of the 4.5 µm by 4.5 µm regions imaged were subjected to vesicle classification; the resulting data are summarized in the table. The number of each type of vesicle relative to the total number of vesicles (elongated and round), at the respective calcium concentrations is given as a percentage in parenthesis. Yellow, pink and green represent elongated, crookedly elongated and round vesicles, respectively. Cream-, straw- and saffron-yellow represent elongated vesicles, tightly elongated vesicles, and tightly elongated vesicles with a crystal-array, respectively.

				Calcium Concentration			
				≤0.9 nM	0.086 µM	0.2 µM	2.0 µM
elongated vesicles *				169 (13.8%)	234 (14.2%)	168 (8.9%)	106 (7.7%)
	tightly elongated			55 (4.5)	100 (6.0)	55 (6.0)	93 (6.8)
		with crystal-array		31 (2.5)	22 (1.3)	0	0
			including tetramer	1 ** (0.08)	0	0	0
		without crystal-array		24 (2.0)	78 (4.7)	55 (2.9)	93 (6.8)
	crookedly elongated			114 (9.3)	134 (8.1)	113 (6.0)	13 (0.9)
round vesicles *				1056 (86.2)	1419 (85.8)	1716 (91.1)	1270 (92.3)
total ***				1225	1653	1884	1376

* Elongated and round vesicles are classified based on the ratio of their major axes to minor axes ≥ 2 and < 2 respectively. ** The image of this tetramer-array vesicle is shown in Figure 3b. *** Significantly agglomerated vesicles were not counted (see text for details).

**Table 2:** Number of elongated and round-types of vesicles, observed at 0.04 nM and 2.0 µM Ca^2+^ in the absence of ATP. The SR vesicles were incubated with each concentration of Ca^2+^ and without ATP at pH 7.4 and 0 °C overnight. For the analysis, 8–13 images were recorded at each calcium concentration (0.04 nM and 2.0 µM Ca^2+^). The regions and populations imaged were not specifically selected, and were different to the regions/populations employed for Table 1. For each concentration, the vesicle populations (84–221) within each of the 4.5 µm by 4.5 µm regions imaged were subjected to vesicle classification; the resulting data are summarized in the table (see text for details). The number of each type of vesicles relative to the total number of vesicles (elongated and round), at the respective calcium concentration is given as a percentage in parenthesis. All of the elongated vesicles observed were crooked and without a crystalline array (Figure 9b,d).

	Calcium Concentration	
	0.02 nM	2.0 µM
Elongated (crookedly) vesicles	47 (3.9%)	65 (2.9%)
round vesicles *	1146 (96.1)	2189 (97.1)
total ***	1193	2254

* Elongated and round vesicles are classified based on the ratio of their major axes to minor axes ≥ 2 and < 2 respectively. *** Significantly agglomerated vesicles were not counted (see text for details).

**Table 3:** Number of elongated and round-type vesicles in SR samples with and without thapsigargin (TG), observed at 0.04 nM Ca^2+^ in the presence of ATP. The SR vesicles (0.3 mg protein/mL) were incubated overnight with and without TG (1.5 µM in DMSO), having 23 mM ATP and 0.04 nM Ca^2+^ at pH 7.4 and 0 °C. The vesicles observed within the electro-microscopic view of 4.5 µm by 4.5 µm were classified as elongated or round types, as described in Table 1. To assess vesicle morphology in the absence of TG, 8 images were recorded from regions that included one or more elongated vesicle with a crystal-array of surface particles (see Figure 10a,b), and the 75–311 vesicles per 4.5 µm by 4.5 µm image were classified. To assess vesicle morphology in the presence of TG, 11 images were recorded from regions that included one or more tightly elongated vesicle(s) without a crystal-array of surface particles (see Figure 10c,d), and the 169–328 vesicles per 4.5 µm by 4.5 µm image were classified. No vesicles with a crystal-array were detected. The data are summarized in the table (see text for details). The number of each type of vesicle relative to the total number of vesicles (elongated and round), at the respective calcium concentration is given as a percentage in parenthesis for both data sets. Yellow, pink and green represent elongated, crookedly elongated and round vesicles, respectively. Cream-, straw- and saffron-yellow represent elongated vesicles, tightly elongated vesicles, and tightly elongated vesicles with a crystal-array, respectively.

			DMSO Only (0.09% (*v*/*v*))	TG
elongated vesicles *			256 (11.9%)	132 (6.3%)
	tightly elongated		221 (10.3)	101 (4.8)
		with crystal-array	8 (0.4)	0 (0)
		without crystal-array	213 (9.9)	101 (4.8)
	crookedly elongated		35 (1.6)	31 (1.5)
round vesicles *			1887 (88.1)	1954 (93.7)
total ***			2143	2086

* Elongated and round vesicles are classified based on the ratio of their major axes to minor axes ≥ 2 and < 2 respectively. *** Significantly agglomerated vesicles were not counted (see text for details).

## Abbreviations

SR	sarcoplasmic reticulum
*E*_1_	high affinity state of Ca^2+^-ATPase for calcium
*E*_2_	low affinity state of Ca^2+^-ATPase for calcium
RyR	ryanodine receptor
EGTA	ethylenebis (oxyethylenenitrilo) tetraacetic acid
TG	thapsigargin
DMSO	dimethylsulfoxide
EM	Electron Microscopy/Microscope
TEM	Transmission Electron Microscopy/Microscope
